# Can novel genetic analyses help to identify low-dispersal marine invasive species?

**DOI:** 10.1002/ece3.1129

**Published:** 2014-06-24

**Authors:** Peter R Teske, Jonathan Sandoval-Castillo, Jonathan M Waters, Luciano B Beheregaray

**Affiliations:** 1 Molecular Ecology Laboratory, School of Biological Sciences, Flinders UniversityAdelaide, South Australia, 5001, Australia; 2 Department of Zoology, University of JohannesburgAuckland Park, 2006, Johannesburg, South Africa; 3 Department of Zoology, University of OtagoPO Box 56, Dunedin, New Zealand

**Keywords:** Ascidian, biological invasion, coalescent theory, founder effect, genetic bottleneck, microsatellites, sea squirt

## Abstract

Genetic methods can be a powerful tool to resolve the native versus introduced status of populations whose taxonomy and biogeography are poorly understood. The genetic study of introduced species is presently dominated by analyses that identify signatures of recent colonization by means of summary statistics. Unfortunately, such approaches cannot be used in low-dispersal species, in which recently established populations originating from elsewhere in the species' native range also experience periods of low population size because they are founded by few individuals. We tested whether coalescent-based molecular analyses that provide detailed information about demographic history supported the hypothesis that a sea squirt whose distribution is centered on Tasmania was recently introduced to mainland Australia and New Zealand through human activities. Methods comparing trends in population size (Bayesian Skyline Plots and Approximate Bayesian Computation) were no more informative than summary statistics, likely because of recent intra-Tasmanian dispersal. However, IMa2 estimates of divergence between putatively native and introduced populations provided information at a temporal scale suitable to differentiate between recent (potentially anthropogenic) introductions and ancient divergence, and indicated that all three non-Tasmanian populations were founded during the period of European settlement. While this approach can be affected by inaccurate molecular dating, it has considerable (albeit largely unexplored) potential to corroborate nongenetic information in species with limited dispersal capabilities.

## Introduction

Marine biological invasions pose considerable evolutionary, ecological, and economic consequences (Grosholz 2002; Bax et al. 2003; Molnar et al. 2008). Even though the problem is well recognized, the number of non-native species arriving in new habitats as a consequence of human activities such as shipping and aquaculture operations continues unabatedly (Molnar et al. 2008; Occhipinti-Ambrogi and Galil 2010). This not only increases the chances of potential invaders establishing themselves but also increases the risk of intraspecific hybridization among successively introduced propagules from different localities in the species' native range, which can increase invasive success because the increased genetic variation makes adaptive evolution more likely (Ellstrand and Schierenbeck 2000). Global climate change exacerbates this trend, not only by facilitating the invasion of habitats by human-introduced species that were previously unable to establish large populations (Diederich et al. 2005; Thuiller et al. 2007) but also by driving poleward range shifts in numerous regions (Ling 2008; Pitt et al. 2010), making it ever more difficult to distinguish between natural and anthropogenic introductions.

The early detection of non-indigenous species increases the chances of eradicating them before they can fully establish themselves (Bax et al. 2001; Lodge et al. 2006), but it is often difficult to distinguish between native and recently established populations of marine species because of the lack of systematic, biogeographic, and historical data (Carlton 2009). Such uncertainty can present major challenges for managers who must prioritize management of unwanted species.

In marine ecosystems, populations of introduced species can sometimes be characterized by their ecological association with disturbed or artificial habitats (particularly in harbors, which present the most likely points of introduction; Carlton and Geller 1993) and by life histories that are conducive to dispersal by means of anthropogenic vectors (e.g., attachment to ships' hulls, or transport in ballast water; Lacoursière-Roussel et al. 2012). However, it is often difficult to rule out the alternative explanation that populations in question represent previously overlooked native taxa (e.g., Teske et al. 2011a). In such cases, genetic information is often considered a particularly powerful means of conclusively identifying a non-native organism.

The most frequently applied criteria for identifying introduced species include (a) large geographic distances between the ranges of potentially introduced populations and their closest relatives (i.e., their likely source populations; Carlton and Geller 1993), which is particularly compelling in the case of trans-oceanic invasions (Geller et al. 2010); and (b) relatively low genetic diversities in non-native versus native populations, reflecting founder effects in the former (Roman and Darling 2007). Unfortunately, there are many examples in which neither of these two criteria can be used to reliably diagnose invasions. First, many introductions do not involve interoceanic transport, but meso-scale colonization events (often following a stepping-stone pattern) into habitats that a particular species can theoretically reach without an anthropogenic vector (Hassan et al. 2003; Golani et al. 2007). Second, genetic diversity indices of recently established populations can be comparatively high when these originated from multiple, genetically differentiated sources (Roman 2006), although this is readily recognizable when these are represented by genetically distinct lineages (Pérez-Portela et al. 2013). Third, in taxa that lack a long-lived dispersal phase, it is difficult to distinguish between natural and recently established populations on the basis of genetic diversity even when the latter originated from a single source. Recruitment in these low-dispersal species is predominantly local such that each site in the native range has a unique combination of alleles (Teske et al. 2011b), and dispersal over greater distances is often only possible by means of rafting (e.g., association with floating objects such as wood or seaweed; Thiel and Gutow 2005). The number of individuals that establish populations at new sites (or at recently depleted sites within the native range) may be so low that these retain only a small portion of their source population's genetic diversity. This apparently makes natural colonization of sites within a species' native range difficult to distinguish genetically from introductions into habitats where the species was not previously represented. It may also explain why only a few studies of species for which historical data were considered unreliable have conclusively indicated that a particular population of low-dispersal marine species is native or introduced (e.g., Turon et al. 2003; Xavier et al. 2009; Stefaniak et al. 2012). This situation is clearly very different from what has been reported for species with high dispersal potential. The latter often show low levels of genetic structure and similar levels of diversity along their native ranges (Kyle and Boulding 2000; Banks et al. 2007) and also between source regions and areas into which they have recently extended their ranges (e.g., Hassan and Bonhomme 2005; Banks et al. 2010).

The study of known or putatively introduced species has until recently been dominated by various approaches of measuring genetic diversity, and tests that determine whether or not a bottleneck has occurred, without providing information on its magnitude and duration.

The fact that the loss of genetic diversity during an introduction is often limited or even absent, particularly when the bottleneck was brief and subsequent population expansion rapid (Carson 1990), or when multiple introductions occurred (Roman 2006), clearly highlights the limitations of such methods. During the past decade, a suite of more sophisticated approaches has been developed that can be used to reconstruct demographic histories in considerable detail. Most of these are Bayesian methods based on coalescent theory (Kingman 1982), and programs in which they are implemented include IMa2 (Hey 2010), DIYABC (Cornuet et al. 2014) and BEAST (Drummond et al. 2012). Although none of these novel methods were developed to specifically distinguish between native and introduced populations, their utility in answering questions about periods of population decline and expansion, and divergence between populations, suggests that they have great potential in uncovering demographic information that can help to inform management decisions.

The necessity of identifying new genetic approaches that can contribute toward resolving the native versus introduced status of low-dispersal species is illustrated by a survey of the recent literature of genetic studies on ascidians (Urochordata, Tunicata, Ascidiacea) (Table 1), a group of sessile marine invertebrates that includes a number of important invasive species (Lambert 2007). Although ascidians have planktonic propagules, their larval duration is so short (e.g., <1 day; Svane and Young 1989) that they must effectively establish themselves in new or depleted habitats by means of a small number of rafting individuals. In the majority of studies, genetic evidence for native versus introduced status of ascidian populations was based on low levels of divergence between the region from which a particular species was first described, and regions from which it has been reported more recently (Table 1). Although several studies have used coalescent-based approaches to study aspects relating to the introduction of ascidians, such as the reconstruction of invasion pathways, there was generally a strong reliance on the accuracy of historical records concerning the study species' native range (e.g., Rius et al. 2012). In these studies, genetic diversity indices were largely inadequate to support the historical data because the native populations rarely had greater diversity than the introduced populations (Table 1). Given the often poor historical records of ascidians coupled with a high incidence of misidentifications and a large number of cryptic species (Carlton 2009; Haydar et al. 2011), this reliance on historical data must be considered problematic.

**Table 1:** The present status of information on native and introduced populations of some widespread ascidians

Species	Native population	Introduced population(s)	Evidence and comments	References
*Asterocarpa humilis*	SW Pacific	South Africa, Chile, NW Europe	Historical records; specimens from Europe are genetically very similar to those from New Zealand	Bishop et al. (2013)
*Botrylloides leachi*	NE Atlantic	SW Pacific, Mediterranean, North America, South Africa	Historical records	Hewitt et al. (2002)
*Botryllus schlosseri*	NE Pacific (but see Berrill 1950)	Europe, N America	Low genetic diversity in Europe (native habitat not sampled); the Indo-Pacific is a center of botryllid diversity	Carlton (2005) and López-Legentil et al. (2006)
*Ciona intestinalis* types A and B	Unresolved	Unresolved	–	Hewitt et al. (2002) and Zhan et al. (2010)
*Clavelina lepadiformis*	Eastern Atlantic	Mediterranean (interior)	Low genetic divergence and high estimates of gene flow between regions	Turon et al. (2003)
*Didemnum vexillum*	NW Pacific(?)	Global	Historical records; samples from Japan had the highest mtDNA diversity, but this was based on a small sample size	Nishikawa (1990) and Stefaniak et al. (2009)
*Herdmania momus*	Indian Ocean, Red Sea	Eastern Mediterranean	Historical records; no clear differences in mtDNA diversity indices between native and most introduced populations	Harant (1927), Pérès (1958) and Rius and Shenkar (2012)
*Microcosmus squamiger*	Australia	Mediterranean/NE Atlantic, southern Africa, New Zealand, India, Japan	Historical records; populations in the native range have higher allelic richness, but the difference is small in some cases (e.g., 4.90 in the NE Atlantic vs. 4.94 in Australia)	Rius et al. (2012)
*Perophora japonica*	NW Pacific	NE Pacific, NE Europe	Historical records	Sanamyan (1998), Streftaris et al. (2005) and Lambert (2005)
*Phallusia nigra*	Red Sea	Indian, Pacific, and Atlantic Oceans	Historical records	Van Name (1945) and Nóbrega et al. (2004)
*Pyura dalbyi*	SE Australia	Western Australia	The Western Australian population is supposedly confined to a small harbor; mtDNA diversity is higher in the introduced population, but this is based on a small sample size	Teske et al. (2011a)
*Pyura praeputialis*	E and SE Australia	Chile	The Chilean population is confined to a single bay; native and introduced populations have similar levels of genetic diversity (based on mtDNA and nrDNA sequence data)	Teske et al. (2011a)
*Styela clava*	NW Pacific	NE and SE Pacific, NW and NE Atlantic	Historical records; populations in the native range have high genetic diversity, but this is not a diagnostic feature to distinguish them from all introduced populations	Goldstien et al. (2011)

**Figure 1:**
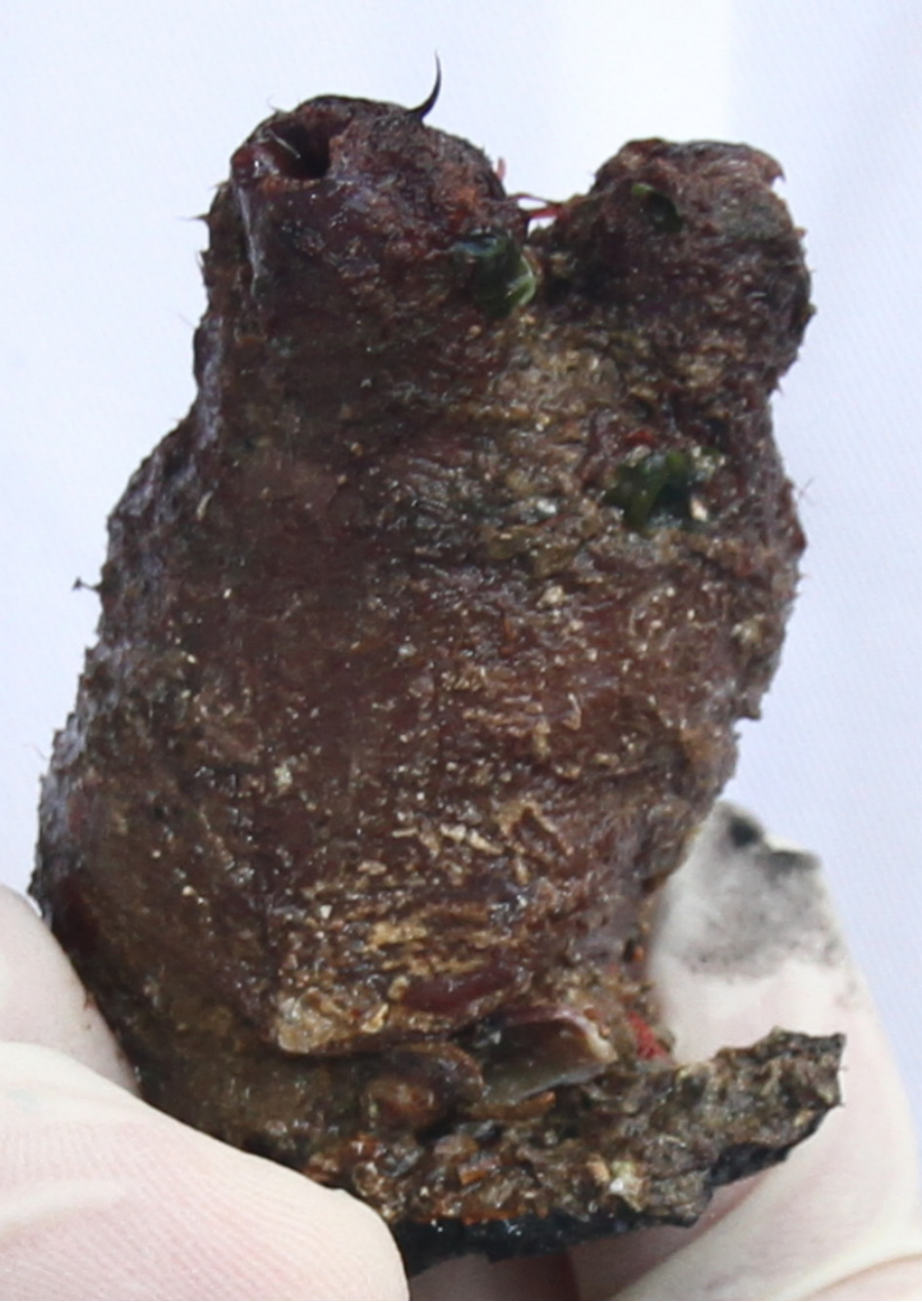
The recently described ascidian *Pyura doppelgangera* Rius and Teske, 2013, is common in Tasmania but rare in mainland Australia and New Zealand. Coalescent-based genetic analyses suggest that this species has recently been introduced to non-Tasmanian localities through human activities.

Here, we explore whether or not coalescent-based methods can be used to distinguish between native and potentially introduced populations of the ascidian *Pyura doppelgangera* Rius and Teske, 2013 (Chordata, Urochordata, Tunicata, Ascidiacea; Fig. 1), a member of the widespread *Pyura stolonifera* species complex, whose species are commonly known as “cunjevoi” or “red bait.” These large, solitary ascidians are common in intertidal and subtidal habitats particularly of the southern hemisphere. They have great potential to become problem species because they are ecosystem engineers that can not only replace native habitat-forming species but they can also provide habitat for other invaders and thus radically alter newly invaded ecosystems (Rius and Teske 2011, 2013). The very localized ranges of *P. doppelgangera* beyond Tasmanian shores (details in Materials and methods), and its presence on artificial structures near harbors in coastal areas of mainland Australia, strongly suggest that all three non-Tasmanian populations (in South Australia, Victoria and New Zealand) may have been recently introduced, most likely through human activities. However, previously generated DNA sequence data from a mitochondrial gene and a nuclear intron (see Fig. S1 and Discussion) have not confirmed this hypothesis (Rius and Teske 2013) and provided conflicting information as a likely consequence of differential levels of lineage sorting. Because of their slow rate of evolution, DNA sequences are typically used to reconstruct a species' demographic history at scales of tens of thousands to millions of years (Avise 2000), making them unsuitable for distinguishing between recent introductions (e.g., by means of anthropogenic vectors since the 19th century, when Pacific trade intensified; Bach 1976) versus natural Holocene colonization scenarios. For this reason, the existence of older, previously overlooked native populations in South Australia, Victoria, and New Zealand cannot be ruled out on the basis of such data.

Microsatellites are appropriate DNA markers for studying near-contemporary demographic events. As their mutation rate is so high that novel mutations are often directly observed in families (Weber and Wong 1993), these markers can provide information at a scale as recent as tens to hundreds of generations (Raeymaekers et al. 2005; Selkoe and Toonen 2006; Nance et al. 2011). We developed a microsatellite library for *P. doppelgangera* (Aksoy et al. 2013) and collected samples from all the regions where this species has been reported (Table 2). We then employed a number of analytical approaches, some of them rarely or not previously applied to this particular research question (including Bayesian Skyline Plots for microsatellite data, Approximate Bayesian Computation and coalescent-based molecular dating), to determine whether there is genetic support for the historical and nongenetic evidence suggesting that the non-Tasmanian populations became established very recently, likely through human activities. Our study pioneers the assessment of the relative power of novel and more traditional genetic approaches to detect recently introduced populations of low-dispersal species, and to differentiate between potentially anthropogenic introductions and ancient divergences, contributing to our understanding of invasion biology in a rapidly changing world.

**Table 2:** Sites at which samples of *Pyura doppelgangera* were collected for this study. All regions from which this species has been reported are represented

Region	Population no.	Population name	Substratum type	Coordinates	Sample size
South Australia^1^	1a	Semaphore Beach	a	34°50′15′′ 138°28′36′′	30
	1b	Grange Beach	a	34°54′09′′ 138°29′14′′	49
	1c	Henley Beach	a	34°55′11′′ 138°29′31′′	45
	1d	Glenelg	a	34°58′50′′ 138°30′35′′	37
W Tasmania	2	Trial Harbor	n	41°55′52′′ 145°10′18′′	28
	3	Couta Rocks	n	41°10′29′′ 144°40′53′′	25
N Tasmania	4	Bridport	a	40°59′26′′ 147°23′27′′	32
E Tasmania	5	The Gardens	n	41°10′25′′ 148°16′52′′	29
	6	Bicheno	a	41°52′12′′ 148°18′12′′	30
	7	Pirates Bay	n	43°01′50′′ 147°56′42′′	26
Victoria	8	Port Welshpool	a	38°42′04′′ 146°27′54′′	20
	9	Port Albert	a	38°40′24′′ 146°41′43′′	30
New Zealand^2^	10a	N Twilight Beach	n	34°29′22′′ 172°40′56′′	3
	10b	S Twilight Beach	n	34°30′32′′ 172°41′59′′	3
	10c	Tauroa Peninsula	n	35°10′12′′ 173°06′22′′	10
	10d	N Herekino	n	35°15′13′′ 173°07′11′′	8
	10e	The Bluff	n	34°41′06′′ 172°53′23′′	9
	10f	Te Werahi Beach	n	34°28′10′′ 172°39′26′′	3
	10g	Tarawamaomao Pt.	n	34°26′12′′ 172°40′30′′	1
Total					418

W, Western; N, North or Northern; E, Eastern; a, artificial; n, natural.

1 The subpopulations comprising population 1 were collected from four geographically proximate jetties in Adelaide, South Australia.

2 Seven geographically proximate sites near the northern tip of Northland, New Zealand.

## Materials and Methods

### Species taxonomy and distribution

Historically, *Pyura doppelgangera* has been synonymized with *P. praeputialis*, a larger species native to eastern and south-eastern Australia. Although morphologically similar, the two species have never been found in sympatry (Rius and Teske 2013), no hybrids were identified using nuclear DNA sequence data (Rius and Teske 2013), and the microsatellite primers developed for *P. doppelgangera* do not cross-amplify in *P. praeputalis* (Aksoy et al. 2013). While the existence of a smaller morph of *P. praeputialis*, whose distribution is centered on Tasmania, has long been known (e.g., Kott 1952; who referred to it as *P. stolonifera*), it has only recently been shown that it is in fact a distinct species (Teske et al. 2011a; Rius and Teske 2013). Additional populations of *P. doppelgangera* have been reported from two localities on the Australian mainland, namely Corner Inlet in Victoria and Adelaide in South Australia (Teske et al. 2011a), and from the northern tip of North Island, New Zealand (Hayward and Morley 2009).

### Sampling and laboratory work

**Figure 2:**
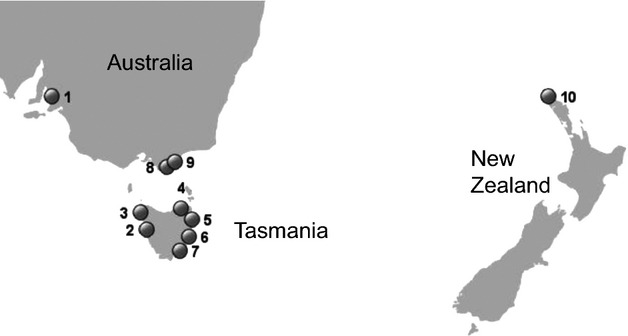
Map of the sampling area. Details on sampling sites 1–10 are given in Table 2. Populations at sites 1, 8, 9, and 10 are potentially non-native.

A total of 418 individuals were collected from six sites in Tasmania, two sites in Victoria, four sites in South Australia, and seven sites in New Zealand (Fig. 2, Table 2). As ascidians collected in close proximity to each other can be expected to be closely related because of a short larval phase and inbreeding (Svane and Young 1989; Dupont et al. 2009), we attempted to sample over as wide a range at a particular site as possible, and pooled samples from geographically proximate sites (in South Australia and New Zealand) for most analyses. Extractions were performed as previously described (Teske et al. 2011a). We used eight of the 10 microsatellite loci developed for *P. doppelgangera* (Aksoy et al. 2013). While this is a comparatively low number of markers, it has been shown to be adequate in previous studies on ascidians, as different populations of these low-dispersal species tend to be highly distinct (Dupont et al. 2009; Zhan et al. 2010; Rius et al. 2012; Reem et al. 2013). PCRs were conducted as described previously (Aksoy et al. 2013), except that the same 58–50°C touchdown protocol (Beheregaray et al. 2004) was used for all subsequent genotyping reactions. The same eight control samples were included in all reactions to ensure that electropherogram peaks were identical for each PCR containing the same primers. Profiles were examined using GENEMAPPER v4.0 (Applied Biosystems, Foster City, CA), and potential scoring errors and null alleles in the genotypes were assessed using MICRO-CHECKER v2.2.3 (van Oosterhout et al. 2004).

### Population genetic analyses

Departures from Hardy–Weinberg equilibrium (HWE) are the norm in ascidian populations due to localized inbreeding and Wahlund effects (Dupont et al. 2009; Zhan et al. 2010). Therefore, where possible we used methods of analyses that do not assume HWE. In cases where this approach was not feasible, we excluded two loci from the data set (see Results for details). Hereafter, we refer to this six-locus data set as the ‘reduced data set’. Several exploratory analyses and estimations of genetic diversity were performed as outlined in the Appendix.

### Analyses of genetic differentiation and relationships among populations

Populations within a species’ native range can often be assigned to distinct phylogeographic lineages whose ranges are linked to biogeography (Teske et al. 2011a). Introduced populations, on the other hand, while often comprising alleles that are also present in the native habitat, tend to have different allele frequencies (Golani et al. 2007), or a combination of alleles from several regional lineages (Roman 2006). We explored genetic relationships among populations, and their relationship with geography, using both population-level and individual-level analyses.

Tests for genetic structure among pairs of populations were conducted in GenAlEx v6.5 (Peakall and Smouse 2012) using the statistics *G*″_ST_ (Meirmans and Hedrick 2011) and *D*_est_ (Jost 2008). *G*″_ST_ is an unbiased estimator of *F*′_ST_ (*F*_ST_ standardized by the maximum value it can obtain; Hedrick 2005), while *D*_est_ is the unbiased estimator of Jost's (2008) *D* (actual population differentiation). Both statistics are particularly suitable for microsatellite data because they are not affected by the high levels of polymorphism typical of these markers. Significance was tested using 999 permutations.

In addition, we used three approaches that do not incorporate information on each individual's population membership. First, a neighbour-joining (NJ) tree (Saitou and Nei 1987) was constructed in PHYLIP (Felsenstein 1989) from Rousset's â indices among pairs of individuals (Rousset 2000) that were calculated in SPAGeDI (Hardy and Vekemans 2002). Rousset's â index is an individual-level analog of the population-level *F*_ST_/(1 − *F*_ST_) ratio (Rousset 2000). We used the reduced data set in this case. Second, we tested for differentiation among individuals using factorial correspondence analyses (FCA) in GENETIX v4.05 (Belkhir et al. 1996–2004). This multivariate method can be applied to any type of data and is thus particularly suitable for data sets that are potentially affected by departures from HWE or LD, so we applied it to the complete data set. Genetic differentiation among populations, if present, is graphically displayed by plotting individuals in multidimensional space. Third, we used STRUCTURE v2.3.2.1 (Pritchard et al. 2000) to determine the most likely number of distinct genetic clusters (*K*) to which individuals of *P. doppelgangera* could be assigned (reduced data set only). As genetic structure was found among most pairs of sites (see Results) and the data set was thus highly informative, we used the admixture model without location priors and set allele frequencies to be independent among populations, with default settings for all advanced parameters. For each of five replications of a particular value of *K* (1–10), the burnin was set to 10^5^ MCMC replicates, followed by 10^6^ recorded replications. In addition to determining the *K* for which the highest likelihood was determined, we estimated the statistic Δ*K* (Evanno et al. 2005), which selects the value of *K* for which the most rapid increase in likelihood is found for successive values of *K*. Maximum L(*K*) and Δ*K* were both plotted with STRUCTURE HARVESTER (Earl and von Holdt 2012).

### Tests for changes in effective population size

Newly established populations often experience rapid genetic drift in the form of a founder effect, which is analogous to a genetic bottleneck. Such population genetic scenarios are particularly likely for introduced ascidians, in which the colonization of new habitats is likely often achieved via the release of gametes by a few translocated adult specimens. We used five different methods and applied these to the reduced data set to explore whether there were clear differences in population size trends between Tasmanian and non-Tasmanian populations. Three of these, implemented in the programs BOTTLENECK (Piry et al. 1999), *M*-ratio (Garza and Williamson 2001), and MSVAR (Beaumont 1999; Storz et al. 2002), are explained in the online appendix. While BOTTLENECK and *M*-ratio calculate summary statistics that provide evidence of past population declines, MSVAR is a coalescent-based approach that identifies a single major change (increase or decrease) in effective population size. Below, we deal with two more recently developed approaches: Bayesian Skyline Plots and Approximate Bayesian Computation.

#### Bayesian Skyline Plots

Extended Bayesian Skyline Plots (EBSPs; i.e., Bayesian Skyline Plots based on more than one locus) were used to explicitly reconstruct each population's effective population size over time. To our knowledge, this is the first time this method has been used to reconstruct population size trends in an animal at the scale of decades rather than millennia, because until recently, no software was available to construct such plots with microsatellite data. The EBSPs were constructed in BEAST v1.74 (Drummond et al. 2012), and settings were based on recommendations by Chieh-Hsi Wu (BEAST developer, University of Auckland, New Zealand). The site models of the different loci were linked, but the clock models and partition trees were not. For the substitution model, we specified equal rates, linear mutation bias and a two-phase model. For the strict clock model, a mutation rate of 4.0 × 10^−4^ (with a 95% confidence interval of 1.3 × 10^−4^ to 1.3 × 10^−3^) was specified based on the mutation rate estimate of the MSVAR analyses (see Results), as no published rates for ascidians are available. While this estimate was recovered irrespective of the priors specified (Online appendix), the mutation rates estimated by this program are not always reliable (e.g., Faurby et al. 2010). Although we consider this particular estimate to be plausible, we also discuss our results in the light of a different choice of mutation rate (see Discussion). A linear model was specified for the coalescent tree prior, and ploidy was set to autosomal nuclear. Default priors were used for model parameters and statistics, except that the demographic population mean was set to uniform, with an initial value of 2500 and upper and lower bounds of 50,000 and 100, respectively, based on the results of an exploratory BEAST run with a constant size tree prior using a combination of samples from the two sites in Victoria. We specified a chain length of 8 × 10^8^ and a logging frequency of 1 × 10^6^, and ran the program on the BIOPORTAL server (Kumar et al. 2009). Each run was repeated twice with the same settings to ensure that searches converged on similar values. As the pooling of samples from multiple sources can considerably affect the Skyline Plots (Heller et al. 2013), we excluded populations that showed evidence for admixture from these and the following analyses (ABC).

#### Approximate Bayesian Computation

The program DIYABC v2.0 (Cornuet et al. 2014) was used to test different hypotheses concerning the populations' effective population sizes before and after a period of demographic expansion (all populations underwent expansions, see Results). If the non-Tasmanian populations were recently founded, then one would expect these to have undergone severe bottlenecks. In contrast, long-established populations, although undergoing demographic changes, would be expected to have much larger sizes prior to demographic expansion. Although recent natural or human-mediated intra-Tasmanian colonization events are likely, and some habitats may have become depleted and then recolonized from nearby sources, we hypothesized that there would be well-established Tasmanian populations in particularly suitable habitats whose numbers remained comparatively large over long periods of time. DIYABC implements Approximate Bayesian Computation (ABC), a bayesian method in which the posterior distributions of the model parameters of interest are determined by a measure of similarity between observed and simulated data rather than each parameter's likelihood (Nielsen and Beaumont 2009). For each population, we determined support for two demographic scenarios: Scenario 1: the effective population size increased from a small number of individuals (1–99) during the past 1000 years, to a larger present population size (100–10,000 individuals); Scenario 2: the same settings were specified, but the starting population size was larger (100–10,000 individuals) but constrained to be smaller than the present population size. Scenario 1 thus represents a founder effect that would be expected if a small number of adults are introduced to a new area by means of a vector (e.g., floating wood or the hull of a ship), while Scenario 2 merely represents an increase in population size. Summary statistics included the mean number of alleles, mean genetic diversity, and mean size variance. Table S1a shows details on priors and mutation models.

### Estimation of the times when populations were founded

Estimates of the time at which a population split from its sister population or underwent a demographic expansion can provide information on whether such demographic events were likely natural or anthropogenic. *Pyura doppelgangera* is believed to have been introduced to New Zealand as recently as a decade ago (Hayward and Morley 2009), and a divergence time estimate that considerably predates this would support a natural introduction hypothesis. This would particularly be the case if it predated the 19th century, during which Pacific trade intensified (Bach 1976) and the likelihood of human-mediated introductions increased considerably. We hypothesized that all non-Tasmanian populations were founded comparatively recently, reflecting recent anthropogenic introductions. Given that older Tasmanian populations could have readily established new populations elsewhere in Tasmania by means of natural or anthropogenic longshore dispersal, and adjacent sites in particular could have maintained some genetic connectivity, we expected that divergence time estimates between Tasmanian populations would not be significantly older than those between Tasmanian and non-Tasmanian populations.

#### IMa2

The program IMa2 (Hey 2010) was used to estimate divergence times between the three non-Tasmanian and the Tasmanian populations. This program uses coalescent theory (Kingman 1982) to jointly estimate the effective population sizes for extant populations and their shared ancestor, and post-divergence migration rates, in addition to divergence time. We estimated these parameters between all possible pairs of Tasmanian populations, and between the non-Tasmanian populations and a combination of data from two Tasmanian populations representing their sister lineage (see Results). We used a geometric heating scheme with two arguments for all runs and converted the scaled divergence time parameter *t* to time in years by specifying a generation time of 1 year and a mutation rate of 5.0 × 10^−4^ mutations per allele per generation. Although it has been reported that the congener *Pyura stolonifera* can reach maturity in as little as 6–10 months (Fielding 1990–1993), we selected a generation time of 1 year to account for periods of low growth, for example during winter. Following a number of test runs to explore trends in the estimation of demographic parameters, a unique upper bound that exceeded each demographic parameter's highest value of the posterior density plots was specified for each data set (Table S2), and 500,000 genealogies were discarded as burn-in, as examination of trend plots indicated that sampled genealogies were independent of the random starting state beyond this point. Each divergence time estimate represents the mean of three independent runs with different heating schemes and starting seeds for which Effective Sample Size (ESS) values (the number of independent points that have been sampled for each parameter, which is an indication of how well independent chains in the simulation have mixed) were particularly high.

#### DIYABC

We used DIYABC to compare three hypotheses that differ only in terms of the timing of an increase in population size. Scenario 1 (recent): 1–49 years; Scenario 2 (historical): 50–399 years; Scenario 3 (prehistoric): 400–1000 years (see Table S1b for details).

## Results

Most loci exhibited significant departures from Hardy–Weinberg proportions, but none did so consistently for all populations (e.g., locus 6: eight out of the total of 10 populations; locus 2: six populations; locus 7: four populations; Table S3). Given that there was no evidence for null alleles or other genotyping problems on the basis of MICRO-CHECKER analyses, we decided to exclude only locus 6 from analyses that assume HWE. Also, as loci 4 and 8 were linked in 8 out of 10 populations (Table S3) we excluded the less variable locus 8 from analyses that assume no LD.

Allelic richness (AR) was higher at Tasmanian sites than at non-Tasmanian sites (Mann–Whitney *U*-test; complete and reduced data set: *P* < 0.05), although this difference was minor in several cases (Table 3). No clear difference between the two groups was found on the basis of Private Allelic Richness (PAR, a measure of how many alleles are unique to a particular population; *U*-test, *P* = 0.2 and *P* = 0.07 for the complete and reduced data sets, respectively), *H*_*O*_ (randomization test; *P* = 0.05 and *P* = 0.10) and the inbreeding coefficient *F*_IS_ (randomization test, *P* = 0.60 and *P* = 0.59), with the highest and lowest values of *F*_IS_ found at non-Tasmanian sites. With the exception of site 1, *F*_IS_ was positive in all populations, indicating heterozygote deficiencies as a possible consequence of nonrandom mating.

**Table 3:** Population genetic summary statistics for *Pyura doppelgangera* microsatellite data at 10 sites (Tasmanian: 2–7; non-Tasmanian: 1, 8–10)

Data set	Site	AR	PAR	*H*_*O*_	*H*_*E*_	*F*_IS_
Complete	**1**	**2.1**	**0.2**	**0.3**	**0.3**	**−0.1**
	2	5.6	1.1	0.5	0.7	0.2
	3	3.5	0.0	0.3	0.4	0.3
	4	3.1	0.1	0.3	0.4	0.3
	5	3.5	0.4	0.4	0.5	0.2
	6	4.2	0.3	0.4	0.5	0.2
	7	5.4	0.3	0.3	0.5	0.4
	**8**	**2.8**	**0.0**	**0.6**	**0.6**	**0.0**
	**9**	**2.8**	**0.1**	**0.3**	**0.4**	**0.3**
	**10**	**2.5**	**0.1**	**0.1**	**0.4**	**0.7**
Reduced	**1**	**2.0**	**0.3**	**0.3**	**0.3**	**−0.1**
	2	5.6	1.2	0.6	0.6	0.2
	3	3.4	0.1	0.3	0.3	0.3
	4	3.1	0.2	0.3	0.3	0.3
	5	3.5	0.5	0.4	0.4	0.2
	6	4.0	0.3	0.4	0.5	0.2
	7	5.2	0.4	0.3	0.5	0.4
	**8**	**2.7**	**0.0**	**0.7**	**0.6**	**0.0**
	**9**	**2.7**	**0.0**	**0.3**	**0.4**	**0.3**
	**10**	**2.0**	**0.1**	**0.1**	**0.4**	**0.7**

The complete data set included all eight microsatellites, while the reduced data set excluded loci 6 and 8. Non-Tasmanian sites are shown in bold.

Acronyms: AR, allelic richness; PAR, private allelic richness, *H*_*O*_, observed heterozygosity; *H*_*E*_ expected heterozygosity; *F*_IS_, inbreeding coefficient.

### Genetic differentiation and relationships among populations

All populations were distinct from one another on the basis of both *G*′′_ST_ and *D*_est_ (*P* < 0.01), except for pairwise comparisons of two adjacent sites in South Australia (sites 1a and 1c) and the two sites in Victoria (sites 8 and 9; *P* > 0.05; Table S4).

**Figure 3:**
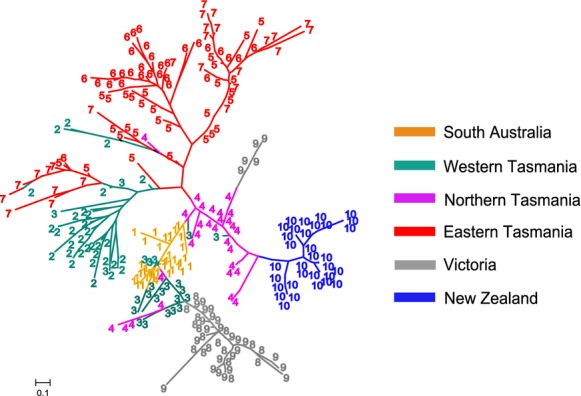
An unrooted neighbour-joining tree constructed from Rousset's â indices between individuals of *Pyura doppelgangera*. Site numbers are the same as those used in Fig. 2 and Table 2.

A neighbour-joining (NJ) tree constructed from Rousset's â indices between individuals (Fig. 3) recovered only the population from New Zealand as a monophyletic lineage. Two Victorian lineages were identified (of which the less common one was only present at site 9), and samples from South Australia clustered among individuals from western and northern Tasmania. None of the potentially introduced non-Tasmanian populations was closely related to individuals from the Tasmanian east coast.

In the FCA plot (Fig. S2), several regional grouping were discernible, with sites 1 (South Australia) and 2 (Western Tasmania) being particularly distinct. However, all groupings showed some overlap.

**Figure 4:**
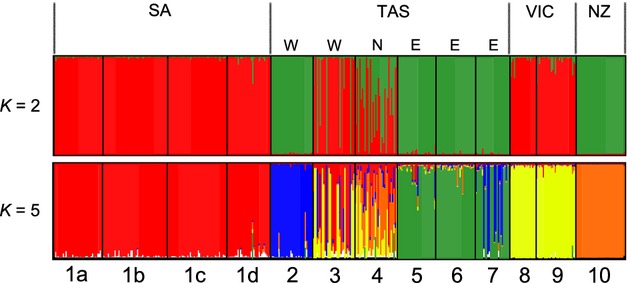
Bar plots depicting the assignment of individuals of *Pyura doppelgangera* from 10 sites to a specified number of clusters (*K*). Each individual is represented by a thin vertical bar. Geographic regions are shown on top, the number of genetic clusters (*K*) on the left, and population numbers (see Table 2) below (SA, South Australia; TAS, Tasmania; VIC, Victoria; NZ, New Zealand; W, west; N, north; E, east).

The most rapid increase in likelihood (Δ*K*) for the number of genetic clusters in STRUCTURE was found for *K* = 2 (Fig. S3). A bar plot for this value of *K* is shown in Fig. 4. The first category (red) for *K* = 2 comprises three non-Tasmanian populations (1, 8, and 9), while the second category (green) comprises Tasmanian populations (2, 5, 6, and 7) and the population from New Zealand (10). The two populations in north-western Tasmania (3 and 4) could not be clearly assigned to either category. Figure 4 also includes a bar plot for *K* = 5. Although this value of *K* was not strongly supported on the basis of Δ*K* (Fig. S3b), its mean L was high (Fig. S3a) and it is included here because it provides additional information at a lower hierarchical level than the plot for *K* = 2. Specifically, it provides information on the putative source populations of the three non-Tasmanian populations, and indicates that all three populations are strongly associated with two north-western Tasmanian populations (sites 3 and 4), a result that is supported by the individual-based NJ tree in Fig. 3. In addition, many of the individuals from site 7 (Eastern Tasmania) have strong affinities with site 2 (Western Tasmania).

### Genetic diversity and population size changes

Wilcoxon tests for heterozygosity excess (indicative of a recent founder event) conducted in BOTTLENECK were nonsignificant for all 10 populations of *P. doppelgangera* (Table S5). Although significant heterozygosity deficiencies (resulting from an excess of low-frequency alleles in populations that have been stable for a long time) were found at several Tasmanian sites, there was no clear distinction between Tasmanian and non-Tasmanian populations. Sites 7 and 9 were excluded from this and several subsequent analyses because results from at least one analysis indicated that these may comprise individuals from different sources. Many individuals at site 7 were assigned to site 2 in Figure 4, and the NJ tree of Rousset's â indices (see Fig. 3) indicated that site 9 comprised two lineages of independent origins. While the latter was not confirmed by the STRUCTURE analysis, excluding site 9 was not considered problematic because its region (Victoria) could be adequately represented by the geographically proximate site 8. The *M*-ratios were estimated for three different models that differed in terms of a priori values specified for the proportion (*p*_s_) and mean size of multi-step mutations (*δ*_g_) (Models 1–3 in Table S6). *M*-ratios were not significant when the most conservative model (Model 1) was applied, and they were all significant when the least conservative model (Model 2) was applied. The application of an intermediate model (as recommended by Peery et al. 2012) resulted in significant *M*-ratios for all except one of the non-Tasmanian populations (site 8).

Reductions in population size were identified with MSVAR for all four data sets, and highest posterior density (HPD) intervals of all mean parameter estimates broadly overlapped (Table S7). In all four cases, the population size changes were estimated to be prehistorical (>1000 years ago). The fact that estimates for the non-Tasmanian populations were similar to those of a population comprising data from all Tasmanian sites indicates that the event resulting in this demographic signature may have affected the species before it split into different regional groups. Estimates for some individual Tasmanian sites also fell within this range (not shown). MSVAR runs had very high ESS values for all parameters (>1200), and results were consistent for multiple runs, suggesting that the program was run for sufficiently long for the priors not to affect demographic estimates.

**Figure 5:**
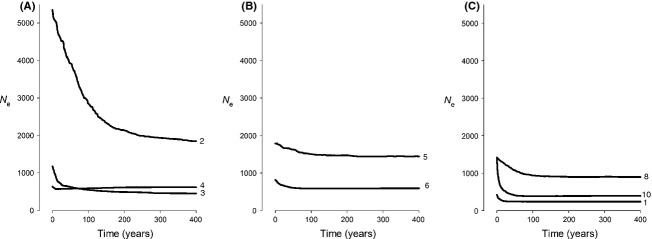
Extended Bayesian Skyline Plots of microsatellite data from eight of the 10 sites at which *Pyura doppelgangera* was collected; (A) eastern Tasmania; (B) northern and western Tasmania; (C) non-Tasmanian sites. Sites 7 and 9 were not included because evidence for a mixed origin suggested that the data from these sites violate the model's assumption that the data from each site represent a single population. To avoid losing resolution, we do not show 95% confidence intervals for *N*_e_, nor do we show events older than 400 years (the oldest occurrence of a change in *N*_e_ at site 2).

Skyline Plots identified expansions in effective population size (*N*_e_) in all populations (Fig. 5), although it was minimal in some cases. Considerable intersite differences were found in initial and final *N*_e_, and in the estimated timing of these expansions, but there were no clear trends that could be used to distinguish between Tasmanian and non-Tasmanian populations. Although one of the Tasmanian populations (site 2) had a signature of an old expansion (∼400 years ago) and showed a gradual increase toward the present to become the largest extant population, the effective sizes of other Tasmanian populations showed expansions as recent as those of some of the non-Tasmanian populations. Very recent population expansions (<10 years ago) were found both at a Tasmanian (site 4) and a non-Tasmanian (site 1) site.

Hypothesis tests performed with DIYABC revealed that, with the exception of the Tasmanian populations 2 and 5, all populations experienced founder effects, with support for a starting population size of less than 100 individuals (Scenario 1 in Table 4a) being particularly strong (≥78%) for samples from the three non-Tasmanian sites (1, 8, and 10). Estimated timings of founder events further supported a recent origin (<50 years; Scenario 1 in Table 4b) for the non-Tasmanian populations, but also for one of the Tasmanian populations (4), while the founder event in the other two Tasmanian populations (3 and 6) occurred during the historical period. There was no support for any founder events that predated the European discovery of Australia *c*. 400 years ago.

**Table 4:** Demographic scenarios supported for populations of *Pyura doppelgangera* on the basis of DIYABC simulations

		Posterior probability (95% CI)		
		
Analysis	Site	Scenario 1	Scenario 2	Scenario 3
(a)	1	**0.80 (0.78–0.82)**	0.20 (0.18–0.22)	
	2	0.38 (0.36–0.40)	**0.62 (0.60–0.64)**	
	3	**0.71 (0.69–0.72)**	0.29 (0.28–0.31)	
	4	**0.58 (0.57–0.60)**	0.42 (0.40–0.44)	
	5	0.22 (0.20–0.23)	**0.78 (0.77–0.80)**	
	6	**0.75 (0.74–0.76)**	0.25 (0.24–0.26)	
	8	**0.78 (0.76–0.79)**	0.22 (0.21–0.24)	
	10	**0.86 (0.84–0.88)**	0.14 (0.12–0.16)	
(b)	1	**0.86 (0.85–0.88)**	0.11 (0.01–0.20)	0.03 (0.02–0.04)
	3	0.31 (0.29–0.33)	**0.41 (0.40–0.43)**	0.28 (0.26–0.30)
	4	**0.52 (0.50–0.53)**	0.20 (0.18–0.22)	0.28 (0.27–0.30)
	6	0.16 (0.14–0.17)	**0.46 (0.45–0.48)**	0.38 (0.37–0.40)
	8	**0.61 (0.60–0.62)**	0.22 (0.20–0.24)	0.17 (0.16–0.18)
	10	**0.81 (0.80–0.82)**	0.09 (0.08–0.09)	0.10 (0.10–0.11)

The best-supported scenario for a particular site is shown in bold. Analysis (a): Comparisons of effective population sizes prior to expansion; scenario 1: 1–99 individuals; scenario 2: 100–10,000 individuals; Analysis (b) (includes only sites for which scenario 1 was supported in the first analysis): Comparisons of the time at which populations were founded; scenario 1 (recent): 1−49 years; scenario 2 (historical): 50–399 years; scenario 3 (prehistorical): 400–1000 years.

### Estimates of divergence times

**Figure 6:**
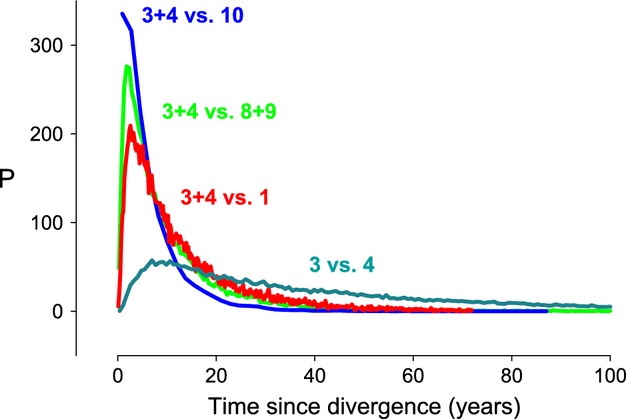
Examples of likelihood plots for divergence time estimates between the four pairs of populations for which the lowest divergence times were estimated. See Table 2 and Fig. 2 for site numbers (*P* = Posterior probability).

The most recent divergence time estimates involved non-Tasmanian populations versus a population comprising combined data from the Tasmanian sites 3 and 4 (the latter were genetically very similar, see Fig. 4) (Table 5; see also Fig. 6 for an example of likelihood plots). Several pairs of Tasmanian populations also had low divergence times that fell within the HPD interval of the former, but these tended to be present among geographically proximate sites that are located in the same biogeographic province (e.g., sites 5 and 6 on the east coast), and upper HPD limits were much larger. Very large divergence times were estimated between N or W Tasmania versus E Tasmania, but also between site 7 and the other E Tasmanian sites. The latter is a likely consequence of site 7 containing a large proportion of individuals that originated from the west coast (see Fig. 4). These results indicate that even though the non-Tasmanian populations were founded recently and some Tasmanian populations apparently have older demographic histories (which supports the idea that *P. doppelgangera* has been present on this island for longer than elsewhere), there is also evidence for recent founder events within Tasmania.

**Table 5:** Times of divergence ± SD (in years) between (a) non-Tasmanian populations and pooled data from the genetically most similar Tasmanian sites 3 and 4, and (b) pairs of Tasmanian populations estimated in IMa2. 95% highest posterior density intervals are shown in brackets

	1	8 + 9	10
(a)			
3 + 4	4 ± 1 (0–57)	2 ± 0 (0–34)	2 ± 1 (0–26)

	2	3	4	5	6
(b)					
3	14 ± 4 (2–426)				
4	18 ± 5 (1–441)	19 ± 9 (2–163)			
5	102 ± 10 (4–855)	37 ± 14 (3–315)	65 ± 4 (4–399)		
6	309 ± 34 (34–1149)	63 ± 1 (6–364)	14 ± 4 (0–497)	22 ± 4 (0–267)	
7	212 ± 4 (46–948)	138 ± 15 (21–663)	159 ± 4 (27–741)	192 ± 34 (7–1028)	440 ± 55 (11–1482)

All values are means from three independent runs (see Table S8) that differed in terms of heating parameters and starting seeds. Sites 7 and 8 were included in this case because the program accounts for post-divergence migration.

## Discussion

Accurate identification of introduced species is a critical first step when managing marine ecosystems and assessing the evolutionary and ecological consequences of biological invasions (Grosholz 2002; Carlton 2009). This goal can be particularly challenging for many invertebrates because of limited historical data and poorly resolved taxonomy. Here, we have described a situation in which the common approach of identifying an introduced species on the basis of (a) reduced genetic diversity and (b) genetic similarity to a more diverse, geographically disjunct population, is not appropriate. Many marine organisms that disperse naturally by means of rafting can establish new populations in the same way as species introduced anthropogenically, and hence genetic diversity assessment may be insufficient for diagnosing species introductions.

In this study, we have assessed the suitability of a number of recently developed and more traditional genetic methods in answering the question whether or not non-Tasmanian populations of an ecosystem engineer, the intertidal ascidian *P. doppelgangera*, were recently introduced by anthropogenic vectors. While the demographic histories of Tasmanian and non-Tasmanian populations could not be clearly distinguished because of recent intra-Tasmanian dispersal, we have identified methods that can be used to provide independent support for introduction hypotheses derived from nongenetic information. Specifically, the finding that colonization events of non-Tasmanian sites fell into the period of European settlement is not consistent with the idea that these are previously overlooked native populations.

### Assessment of summary statistics

Tests for genetic bottlenecks that produce a simple “yes-or-no answer” failed to distinguish between Tasmanian and non-Tasmanian populations. Specifically, a test for heterozygote excess did not identify any bottlenecks, whereas the *M*-ratio test identified bottlenecks in all populations (except in one that is likely to have been recently introduced on the basis of coalescent-based approaches), although it must be conceded that the low number of samples and loci could have affected the latter result (Peery et al. 2012). Furthermore, a simple coalescent-based approach implemented in MSVAR identified a population reduction at a temporal scale that is clearly inapplicable to the ecological timeframes under consideration here. Of the different methods that can be used to detect reductions in population size indicative of a founder event by means of summary statistics, a clear difference between Tasmanian and non-Tasmanian populations of *P. doppelgangera* was only found using a comparatively simple measure, allelic richness. The three non-Tasmanian populations had the lowest allelic richness, while the highest values were found in Tasmanian populations. However, the utility of this statistic for detecting recent founder events seems limited given that low allelic richness would be expected in any small, isolated population of a low-dispersal marine invertebrate.

### Analyses of genetic differentiation and relationships among populations

Analogs of the population structure statistic *F*_ST_ revealed that most populations of *P. doppelgangera* were distinct, with the exception of adjacent sites in South Australia (site 1) and Victoria (sites 8 and 9). *F*-statistics are often used to identify source populations in species with high dispersal potential; long-established populations from different sites within ‘high-connectivity’ stretches of coast tend to show little or no genetic structure (Banks et al. 2007; Teske et al. 2011a), whereas recently introduced populations are often distinct and differ from the source populations primarily because of their lower genetic diversity (Golani et al. 2007). As genetic structure in low-dispersal species can be described by a pattern of isolation-by-distance along continuous coastlines, with populations at each site being genetically unique (Kyle and Boulding 2000; Hoffman et al. 2013), this approach is of limited use in these cases.

Methods that group individuals on the basis of genetic similarities (FCA, NJ tree and STRUCTURE) suggested that Tasmanian and non-Tasmanian populations were closely related to one another. The NJ tree (Fig. 3) indicated that each of the non-Tasmanian populations had recently derived from lineages resident in western and northern Tasmania. The clustering approach implemented in STRUCTURE showed this pattern even more clearly, suggesting that north-western Tasmania may be the source region of the non-Tasmanian populations. While this finding on its own is unsuitable to identify introduced species because it lacks temporal information, it provides an important starting point for subsequent analyses.

### Bayesian Skyline Plots

Skyline plots are perhaps the most sophisticated approach presently available to assess long-term demographic change, as they provide detailed information on a population's effective population time from the present to the point in the past at which its extant lineages coalesce. However, for the purpose of identifying recent introductions, this approach has several shortcomings. First, while the plots report confidence intervals for the effective population size, *N*_e_ (*y*-axis; not included in Fig. 5 to avoid losing resolution), they do not report confidence intervals for time (*x*-axis), so there is no information about the accuracy of the time estimate at which a population has started to expand. Second, it is impossible to rule out the possibility that the *N*_e_ of a recently founded population prior to expansion contains demographic signal from its source population, particularly when the number of founders was large. Third, even if a population was founded by very few individuals and a low starting population size is identified, skyline plots do not report when the new population split from its ancestor. Instead, the oldest *N*_e_ is reported from the time at which all present-day gene copies coalesce, which can considerably predate the time of divergence between two populations (Arbogast et al. 2002).

### Approximate Bayesian Computation

ABC methods can be used to compare alternative models of great complexity because they do not require the estimation of each demographic parameter's full likelihood (Beaumont 2010). We used this approach to determine support for some comparatively simple one-population models, and posterior probabilities were highest for the hypothesis that all three non-Tasmanian populations were recently founded by a low number of individuals. However, the same was also true for several of the Tasmanian populations, and only the results for populations 2 and 5 suggest that Tasmania is the native habitat of *P. doppelgangera*.

### IMa2

While the scenarios that can be investigated with the full likelihood method IMa2 are often unrealistically simple compared to the scenarios that can be modeled with ABC, a two-population scenario in which one population represents the putative source and the other the introduced species is adequate for studying divergence in *P. doppelgangera*. Estimates of divergence time between the non-Tasmanian populations and a lineage comprising genetic data from the two Tasmanian populations identified as being most closely related on the basis of the STRUCTURE analysis were the most recent among any of the population pairs compared. The HPD intervals were narrow compared to those of pairs of Tasmanian populations, which suggests that these estimates are quite accurate. As was the case for the ABC analyses, we found strong support for a recent origin of the non-Tasmanian populations, but we also found that, on the basis of HPD intervals, none of the Tasmanian population pairs diverged from each other before the historical period (*c*. 400 years ago). This suggests that numerous founder events occurred during this period as a result of (natural or anthropogenic) intra-Tasmanian translocations.

### Suitability of genetic markers to study marine invasions

Despite the low number of microsatellite loci used in the present study (depending on the analyses, as few as six), these data were considerably more powerful for resolving the native versus introduced status of *P. doppelgangera* than were DNA sequence data (mtDNA COI and nuclear ANT). Contradictions between the two sequence markers can be likely ascribed to either incomplete lineage sorting or mtDNA-specific inheritance. For example, we found no evidence for the South Australian population being a long-established sister lineage of western or eastern Tasmanian populations (as indicated by the COI data, which differed by a minimum p-distance of 0.01, suggesting ancient divergence when a mutation rate of ∼1% per million years is applied; Meyer et al. 2005; Fig. S1a), or two independent introductions into South Australia and an east Tasmanian origin of the population in New Zealand (as indicated by the ANT data; Fig. S1b). DNA sequence data, and in particular mitochondrial data, are still by far the most commonly used molecular markers to study invasive species (e.g., Rius and Shenkar 2012; Stefaniak et al. 2012; Torkkola et al. 2013; Pérez-Portela et al. 2013), but their contribution to resolving whether specific populations of *P. doppelgangera* are native or introduced would have been negligible at best and misleading at worst.

As with every method that uses molecular dating, divergence time estimates depend considerably on the mutation rate specified. The mutation rate of microsatellites is typically in the range of 1 × 10^−2^ to 1 × 10^−6^ mutations per locus per generation, with a mean mutation rate of about 5 × 10^−4^ (Schlötterer 2000), although it is usually faster in endotherms (e.g., humans: ∼10^−2^ to 10^−4^; Ellegren 2000) than in ectotherms (e.g., *Cyprinus carpio* (teleost): 5.56 × 10^−4^; Yue et al. 2007). For the IMa2 analyses, we specified a mutation rate of 4.0 × 10^−4^ per generation estimated using MSVAR, while the 95% HPD interval from the MSVAR analysis (1.3 × 10^−4^ to 1.3 × 10^−3^) was used as a prior in the ABC analyses. A mutation rate an order of magnitude slower than the one used here would have resulted in IMa2 time estimates between 20 and 40 years for divergence between the non-Tasmanian populations and their Tasmanian sister population, still well within the period of European settlement. Even a mutation rate one-hundredth of the one used here would have been insufficient to reject the hypothesis that the non-Tasmanian populations were founded sometime during the past 200 years, as all three estimates have a lower 95% HPD limit of zero. The approach of estimating the mutation rate used here is undoubtedly inferior to pedigree-based estimations (e.g., Molnar et al. 2012), and we cannot rule out that ascidians have unusually slow microsatellite mutation rates because no such data are yet available for this group. This, however, is unlikely because their overall rate of genome evolution is actually faster than that of vertebrates (Tsagkogeorga et al. 2012).

Another factor that needs to be considered is that even though the microsatellite molecular clock remains linear for about 10,000 generations (Feldman et al. 1997), and combining microsatellite loci allows for reasonably precise molecular dating (Sun et al. 2009), the accuracy of demographic estimates is directly proportional to the number of genetic markers used (Felsenstein 2006). This suggests that the HPD intervals of population size and divergence time estimates in *P. doppelgangera* would have been narrower if a greater number of loci had been genotyped. This ascidian was a particularly challenging species for which to develop microsatellites, because in addition to its genome containing among the lowest number of microsatellites of 154 eukaryotes processed using the same 454 sequencing approach (reviewed in Meglécz et al. 2012), *P. doppelgangera* also had a comparatively large number of loci that did not amplify consistently or that were invariable (see Aksoy et al. 2013 for details). In most other species, it should be possible to develop a much more substantial microsatellite library with the same sequencing effort. The identification of introductions that occurred at a near-contemporary scale is an example where microsatellites perform adequately and will not likely be completely replaced by SNPs from Next-Generation Sequencing (NGS) approaches in the near future. The considerable number of independent loci represented by SNPs suggests that future studies of marine invasions will be able to pinpoint the timing of an invasion, as well as the number of founder individuals, with considerably greater accuracy. However, the method requires better quality DNA than is typically required for microsatellite genotyping, which is a common problem in marine invertebrates (Toonen et al. 2013). For that reason, NGS approaches will likely remain challenging for numerous invertebrates.

## Conclusions

Distinguishing between natural and anthropogenic introductions is becoming increasingly difficult and important. The life history and meso-scale geographic range of species like *P. doppelgangera* present a challenge to the differentiation of native and introduced populations on the basis of genetic methods, but this example is by no means unusual given the ubiquity of low-dispersal marine animals that naturally establish themselves by means of a few founder individuals. In addition to the ascidians, these include other common marine taxa such as peracarid crustaceans, echinoderms and mollusks (Highsmith 1985; Thiel 2003). We found that methods that compare trends in effective population size, including EBSPs for microsatellites and DIYABC for single populations, are unsuitable to distinguish reliably between native and introduced populations because episodes of low population size following a colonization event are a natural occurrence in low-dispersal species. In contrast, coalescent-based approaches that can provide information about a population's demographic history at a temporal scale that is suitable to detect potential anthropogenic introductions (implemented in the programs DIYABC and IMa2) can be useful to rule out the possibility that a species that is suspected to be a recently introduced alien is actually a long-established, but previously overlooked, native species. In conjunction with historical data, local knowledge and information on whether or not a particular species is primarily represented on artificial structures and in harbors, data from these novel genetic methods can contribute toward making a management decision concerning a marine organism that is suspected to have been recently introduced.

## Data Archiving Statement

Final DNA sequence assembly uploaded as online supplementary material. Microsatellite data uploaded as online supplementary material.

Data available from the Dryad Digital Repository: http://dx.doi.org/10.5061/dryad.s584c.

## Supporting Information

**Table S1.** Priors, mutation models and summary statistics used for DIYABC analyses; (a) comparison of effective population sizes prior to expansion; (b) comparisons of the times at which populations were founded. **Table S2.** Upper bounds specified for demographic parameters in the IMa2 analyses. **Table S3.** Loci that show departures from Hardy–Weinberg Equilibrium (HWE) and departures from Linkage Disequilibrium shown for each of 10 sites at which *Pyura doppelgangera* was collected. **Table S4.** Values of the statistics *G*″_ST_ and *D*_est_ calculated for pairs of sampling sites. **Table S5.** Results of one-tailed Wilcoxon tests conducted on microsatellite data of *Pyura doppelgangera* at 10 sites using the program BOTTLENECK. **Table S6.** *M*-ratios calculated for *Pyura doppelgangera* data from eight sites using three different models. **Table S7.** Mean estimates of four demographic parameters estimated in MSVAR. **Table S8.** Pairwise estimates of divergence time (T) among Tasmanian populations of *Pyura doppelgangera*.

**Figure S1.** Maximum-likelihood bootstrap trees of (a) COI sequences and (b) ANT intron sequences.

**Figure S2.** Factorial correspondence analysis (FCA) plots using microsatellite data from 10 populations of *Pyura doppelgangera* from Tasmania, South Australia, Victoria, and New Zealand.

**Figure S3.** Estimation of the number of clusters (*K*) in the reduced data set of *Pyura doppelgangera* microsatellites.

**Data S1.** Fasta format, *Pyura doppelgangera* ANT intron sequences.

**Data S2.** Fasta format, *Pyura doppelgangera* COI gene sequences.

**Data S3.** Genepop format, *Pyura doppelgangera* microsatellites, 10 populations.

**Data S4.** Genepop format, *Pyura doppelgangera* microsatellites, 10 populations, reduced data set.
