# Supplementary material for: Can novel genetic analyses help to identify low-dispersal marine invasive species?
Source: Ecol Evol. 2014 Jun 24;4(14):2848–66. doi: 10.1002/ece3.1129 (PMC4130444; doi:10.1002/ece3.1129)
Supplement: Supplementary file 1 [file ece30004-2848-SD1.docx]

**Appendix**

*Appendix Methods*

Exploratory population genetic analyses were performed in ARLEQUIN v3.5.1.3 (Excoffier and Lischer 2010) and included estimates of observed and expected heterozygosity (*H*_O_ and *H*_E_, respectively), and tests for linkage disequilibrium (LD) and for departures from HWE. Exact tests for departures from HWE (Guo and Thompson 1992) included 10^6^ steps in the Markov chain and 10^5^ dememorisation steps, and tests for LD (Slatkin and Excoffier 1996) were based on 10^3^ permutations and two initial conditions for the EM (Expectation-Maximisation) algorithm. Allelic richness (AR, the number of alleles in a sample) and private allelic richness (PAR, i.e. the number of alleles present at a particular site that were not present at any other site; Kalinowski 2004) were calcuated using HP-Rare v1.1 (Kalinowski 2005). Rarefaction was applied to account for differences in sample sizes (20 individuals, Table 1). Differences between groups comprising Tasmanian versus non-Tasmanian sites were tested using the Mann-Whitney *U* test. For each site, we also calculated the inbreeding coefficient (*F*_IS_) using FSTAT v2.9.3. To test for significant differences in *H_O_* and *F*_IS_ between groups comprising Tasmanian versus non-Tasmanian sites, we conducted two-sided randomisation tests with 1000 permutations in FSTAT. The program CREATE v1.37 (Coombs et al. 2008) was used to convert the microsatellite data file in GENEPOP v3.4 (Raymond and Rousset 1995) format into the formats required by other programs.

a) BOTTLENECK

The program BOTTLENECK v1.2.02 (Piry *et al.* 1999) was used to test whether the gene diversity of the potentially bottlenecked population is greater than the expected equilibrium gene diversity that is computed from the observed number of alleles (Luikart et al. 1998). A two-phase mutation model (TPM; Di Rienzo et al. 1994) is considered to be more adequate for microsatellite data (Ellengren, 2000) than either the pure infinite allele model (IAM; Kimura and Crow, 1964) or the stepwise mutation model (SMM; Ohta and Kimura 1973). Following Piry et al. (1999), we tested for the significance of potential bottlenecks with a Wilcoxon signed-rank test under a TPM (with the default variance of 30) with 95% SMM and 5% IAM for 5000 iterations.

b) *M*-ratio

We used the *M*-ratio test (Garza and Williamson 2001) to identify past population declines on the basis of the ratio (*M)* of the number of microsatellite alleles of a particular population and the range in allele size. In recently founded populations, this ratio is assumed to be reduced. Results of the *M*-ratio test depend considerably on the specified values for the proportion (*p*_s_) and mean size of multi-step mutations (δ_g_), information is not actually available for most species. Following Peery *et al.* (2012), we specified *p*_s_ = 0.22 and δ_g_ = 3.1, but we also compared the results obtained with these settings with the results based on more conservative and less conservative settings (more conservative: *p*_s_ = 0.88, δ_g_ = 3.1; less conservative: *p*_s_ = 0.1, δ_g_ = 2.8). The population size parameter  (4*N*_e_μ, where *N*_e_ is the effective population size and  the locus-specific mutation rate) was estimated for each population using the program IMa2 (Hey 2010), and  was calculated with MSVAR v1.3 (Beaumont 1999; Storz et al. 2002).

c) MSVAR

The program MSVAR v1.3 uses a Markov chain Monte Carlo (MCMC) method to sample from the posterior distribution of four model parameters, namely current effective population size, ancestral effective population size prior to a change in population size, the time at which the population size change occurred, and the mutation rate of the microsatellite loci. A decrease from ancestral to currective effective population size indicates that a founder event may have occurred. We ran four different data sets in MSVAR: one each for the non-Tasmanian populations (the two populations from Victoria were pooled because they were not genetically distinct, see Results), and a combination of all Tasmanian populations. A generation time of 1 year was specified, and the same starting priors were used for all data sets and loci: population size (both past and present, so as not to favour expansion over decline) = 10 000 individuals, mutation rate = 5.0 x 10^-4^ per year, and time since exponential population size change = 1000 years, while default values were specified for all other settings. We specified a thinning interval of 1.0 x 10^6^ steps and ran the program on Flinders University’s Colossus supercomputer for one week, after which runs were automatically aborted. This resulted in between ~9 000 and ~23 000 saved steps for the largest and smallest data-sets, respectively. Results for the first run performed for each data set are shown in Appendix Table 6. Each run was repeated with different starting seeds to ensure consistency of results, and a third run was performed with all starting priors set to 1/10 of the original values. Results were very similar for these three runs, with mutation rate estimates even being identical to the fifth decimal number. Together with the fact that there were no trends in likelihood values when these were examined in Tracer v1.5 (available at http://tree.bio.ed.ac.uk/software/tracer/), following a burn-in of 10% of saved steps in the Markov chain that were discarded, this suggested that the program was run for sufficiently long for priors not to affect the demographic estimates.

| **Appendix Table 1** Priors, mutation models and summary statistics used for DIYABC analyses; a) comparison of effective population sizes prior to expansion; b) comparisons of the times at which populations were founded. | | | | | |
| --- | --- | --- | --- | --- | --- |
| a) | Models | Model 1:  bottleneck | Model 2:  size increase |  |  |
|  |  | N1 | N1 |  |  |
|  |  | 0 sample 1 | 0 sample 1 |  |  |
|  |  | t1-db Var Ne N1d | t1 VarNe 1 Ns |  |  |
|  |  | t1 Var Ne 1 Na |  |  |  |
|  |  |  |  |  |  |
|  | Priors | Parameter | Condition | Min | Max |
|  |  | N1 |  | 10 | 10000 |
|  |  | t1 |  | 1 | 1000 |
|  |  | db | db < t1 | 1 | 5 |
|  |  | N1d | N1d < N1 | 1 | 99 |
|  |  | Ns | Ns < N1 | 10 | 10000 |
|  |  | Na |  | 10 | 10000 |
|  |  |  |  |  |  |
|  | Mutation model | Models | Prior distribution | Min | Max |
|  |  | Mean mutation rate | Uniform | 1.30E-04 | 1.30E-03 |
|  |  | Indiv. Locus mut rate | Gamma | 1.30E-05 | 1.30E-02 |
|  |  | Mean coeff P | Uniform | 1.00E-01 | 3.00E-01 |
|  |  | Ind loc coeff P | Gamma | 1.00E-02 | 9.00E-01 |
|  |  | Mean SNI rate |  | 0 | 0 |
|  |  | Ind loc SNI rate |  | 0 | 0 |
|  |  | Mean mutation rate | Uniform | 1.30E-04 | 1.30E-03 |
|  |  |  |  |  |  |
|  |  |  |  |  |  |
| b) | Models | Model 1:  recent | Model 2:  historical | Model 3: prehistorical |  |
|  |  | N1 | N1 | N1 |  |
|  |  | 0 sample 1 | 0 sample 1 | 0 sample 1 |  |
|  |  | t1-db Var Ne 1 N1d | t2-db VarNe 1 N1d | t2-db VarNe 1 N1d |  |
|  |  | t1 VarNe 1 Na | t2 VarNe 1 Na | t2 VarNe 1 Na |  |
|  |  |  |  |  |  |
|  | Priors | Parameter | Condition | Min | Max |
|  |  | N1 |  | 10 | 10000 |
|  |  | t1 |  | 1 | 49 |
|  |  | t2 | t2 > t1 | 50 | 399 |
|  |  | t3 | t3 > t2 | 400 | 1000 |
|  |  | Db | db < t1 | 1 | 5 |
|  |  | N1d | N1d < N1 | 1 | 99 |
|  |  | Na |  | 10 | 10000 |
|  |  |  |  |  |  |
|  | Mutation model | Models | Prior distribution | Min | Max |
|  |  | Mean mutation rate | Uniform | 1.30E-04 | 1.30E-03 |
|  |  | Indiv. Locus mut rate | Gamma | 1.30E-05 | 1.30E-02 |
|  |  | Mean coeff P | Uniform | 1.00E-01 | 3.00E-01 |
|  |  | Ind loc coeff P | Gamma | 1.00E-02 | 9.00E-01 |
|  |  | Mean SNI rate |  | 0 | 0 |
|  |  | Ind loc SNI rate |  | 0 | 0 |
|  |  | Mean mutation rate | Uniform | 1.30E-04 | 1.30E-03 |
|  |  |  |  |  |  |
|  |  |  |  |  |  |

| **Appendix Table 2** Upper bounds specified for demographic parameters in the IMa2 analyses; t = population splitting parameter; _0_ = effective population size at Site 0; _1_ = effective population site at Site 1; _2_ = population size of the shared ancestor; m0>m1: gene flow from Site 0 to Site 1; m1>m0: gene flow from Site 1 to Site 0. | | | | | | | |
| --- | --- | --- | --- | --- | --- | --- | --- |
| Site 0 | Site 1 | t | _0_ | _1_ | _2_ | m0>m1 | m1>m0 |
| 3+4 | 1 | 0.12 | 1 | 0.2 | 100 | 100 | 100 |
| 3+4 | 8+9 | 0.12 | 1 | 0.4 | 100 | 100 | 100 |
| 3+4 | 10 | 0.12 | 1 | 0.25 | 100 | 100 | 100 |
|  |  |  |  |  |  |  |  |
| 2 | 3 | 0.5 | 3 | 1 | 150 | 50 | 250 |
|  | 4 | 0.5 | 3 | 1 | 150 | 50 | 100 |
|  | 5 | 0.6 | 5 | 3 | 250 | 50 | 50 |
|  | 6 | 1.2 | 4 | 4 | 600 | 750 | 750 |
|  | 7 | 0.5 | 3 | 3 | 150 | 50 | 50 |
| 3 | 4 | 0.3 | 1 | 1 | 150 | 500 | 150 |
|  | 5 | 0.3 | 1 | 1 | 150 | 75 | 125 |
|  | 6 | 1.2 | 4 | 4 | 600 | 750 | 750 |
|  | 7 | 0.6 | 1 | 3 | 300 | 50 | 50 |
| 4 | 5 | 1.2 | 2 | 3 | 600 | 300 | 300 |
|  | 6 | 1.2 | 4 | 4 | 600 | 750 | 750 |
|  | 7 | 0.6 | 1 | 3 | 300 | 100 | 25 |
| 5 | 6 | 1.2 | 4 | 4 | 600 | 750 | 750 |
|  | 7 | 1 | 1 | 4 | 600 | 300 | 50 |
| 6 | 7 | 1.2 | 4 | 4 | 600 | 750 | 750 |
|  |  |  |  |  |  |  |  |

*Appendix Results*

| **Appendix Table 3** Loci that show departures from Hardy-Weinberg Equilibrium (HWE) and departures from Linkage Disequilibrium shown for each of 10 sites at which *Pyura doppelgangera* was collected. | | |
| --- | --- | --- |
| Population | Loci not in HWE | Linked loci |
| 1 | 2,3,6,7 | 4 + 8 |
| 2 | 2,6 | 2 + 6, 4 + 8 |
| 3 | 1,3,6,7 | 1 + 3-8, 2 + 4, 2 + 8, 3 + 4-8, 4 + 5, 4 + 8, 5 + 6-8, 6 + 7, 7 + 8 |
| 4 | 6,7 | 4 + 6,4 + 8, 6 + 8 |
| 5 | 2,4,8 | 1 + 7, 4 + 8, 6 + 8 |
| 6 | 2,5 | 2 + 3, 4 + 8 |
| 7 | 2,4,5,6,8 | 2 + 5, 4 + 8 |
| 8 | 6 | None |
| 9 | 5,6,7 | 1 + 6 |
| 10 | 2,4,5,6,8 | 2 + 4, 4 + 5, 4 + 6, 4 + 8, 5 + 6, 6 + 8 |
| Numbers used for loci are: 1 = *Pysp*02, 2 = *Pysp*03, 3 = *Pysp*12, 4 = *Pysp*13, 5 = *Pysp*15, 6 = *Pysp*19, 7 = *Pysp*25, 8 = *Pysp*26. See Table 2 and Fig. 2 for numbers representing populations. | | |

| **Appendix Table 4** Values of the statistics *G*_ST_ and *D*_est_ calculated for pairs of sampling sites. Values in bold indicate comparisons that had *P*-values > 0.05. | | | | | | | | | | | | | | |
| --- | --- | --- | --- | --- | --- | --- | --- | --- | --- | --- | --- | --- | --- | --- |
|  |  | 1a | 1b | 1c | 1d | 2 | 3 | 4 | 5 | 6 | 7 | 8 | 9 | 10 |
| *G*_ST_ | 1a |  | 0.019 | **0.574** | 0.013 | <0.01 | <0.01 | <0.01 | <0.01 | <0.01 | <0.01 | <0.01 | <0.01 | <0.01 |
|  | 1b | 0.022 |  | 0.047 | 0.019 | <0.01 | <0.01 | <0.01 | <0.01 | <0.01 | <0.01 | <0.01 | <0.01 | <0.01 |
|  | 1c | **0.000** | 0.012 |  | <0.01 | <0.01 | <0.01 | <0.01 | <0.01 | <0.01 | <0.01 | <0.01 | <0.01 | <0.01 |
|  | 1d | 0.041 | 0.026 | 0.035 |  | <0.01 | <0.01 | <0.01 | <0.01 | <0.01 | <0.01 | <0.01 | <0.01 | <0.01 |
|  | 2 | 0.762 | 0.752 | 0.762 | 0.685 |  | <0.01 | <0.01 | <0.01 | <0.01 | <0.01 | <0.01 | <0.01 | <0.01 |
|  | 3 | 0.553 | 0.536 | 0.574 | 0.487 | 0.611 |  | <0.01 | <0.01 | <0.01 | <0.01 | <0.01 | <0.01 | <0.01 |
|  | 4 | 0.703 | 0.694 | 0.717 | 0.609 | 0.441 | 0.283 |  | <0.01 | <0.01 | <0.01 | <0.01 | <0.01 | <0.01 |
|  | 5 | 0.850 | 0.851 | 0.860 | 0.792 | 0.503 | 0.677 | 0.497 |  | <0.01 | <0.01 | <0.01 | <0.01 | <0.01 |
|  | 6 | 0.925 | 0.922 | 0.931 | 0.881 | 0.554 | 0.736 | 0.526 | 0.168 |  | <0.01 | <0.01 | <0.01 | <0.01 |
|  | 7 | 0.880 | 0.886 | 0.890 | 0.835 | 0.441 | 0.717 | 0.563 | 0.196 | 0.275 |  | <0.01 | <0.01 | <0.01 |
|  | 8 | 0.780 | 0.784 | 0.803 | 0.756 | 0.837 | 0.370 | 0.560 | 0.707 | 0.749 | 0.774 |  | **0.058** | <0.01 |
|  | 9 | 0.803 | 0.810 | 0.825 | 0.778 | 0.823 | 0.407 | 0.565 | 0.685 | 0.731 | 0.755 | **0.028** |  | <0.01 |
|  | 10 | 0.891 | 0.888 | 0.899 | 0.839 | 0.667 | 0.702 | 0.511 | 0.530 | 0.595 | 0.677 | 0.700 | 0.689 |  |
|  |  |  |  |  |  |  |  |  |  |  |  |  |  |  |
| *D*_est_ | 1a |  | 0.020 | **0.576** | 0.013 | <0.01 | <0.01 | <0.01 | <0.01 | <0.01 | <0.01 | <0.01 | <0.01 | <0.01 |
|  | 1b | 0.005 |  | 0.047 | 0.018 | <0.01 | <0.01 | <0.01 | <0.01 | <0.01 | <0.01 | <0.01 | <0.01 | <0.01 |
|  | 1c | **0.000** | 0.002 |  | <0.01 | <0.01 | <0.01 | <0.01 | <0.01 | <0.01 | <0.01 | <0.01 | <0.01 | <0.01 |
|  | 1d | 0.010 | 0.006 | 0.008 |  | <0.01 | <0.01 | <0.01 | <0.01 | <0.01 | <0.01 | <0.01 | <0.01 | <0.01 |
|  | 2 | 0.559 | 0.538 | 0.551 | 0.473 |  | <0.01 | <0.01 | <0.01 | <0.01 | <0.01 | <0.01 | <0.01 | <0.01 |
|  | 3 | 0.267 | 0.246 | 0.274 | 0.227 | 0.423 |  | <0.01 | <0.01 | <0.01 | <0.01 | <0.01 | <0.01 | <0.01 |
|  | 4 | 0.414 | 0.392 | 0.419 | 0.328 | 0.271 | 0.127 |  | <0.01 | <0.01 | <0.01 | <0.01 | <0.01 | <0.01 |
|  | 5 | 0.673 | 0.668 | 0.684 | 0.592 | 0.352 | 0.477 | 0.302 |  | <0.01 | <0.01 | <0.01 | <0.01 | <0.01 |
|  | 6 | 0.785 | 0.771 | 0.793 | 0.698 | 0.368 | 0.505 | 0.291 | 0.081 |  | <0.01 | <0.01 | <0.01 | <0.01 |
|  | 7 | 0.732 | 0.736 | 0.744 | 0.663 | 0.300 | 0.529 | 0.365 | 0.111 | 0.144 |  | <0.01 | <0.01 | <0.01 |
|  | 8 | 0.474 | 0.467 | 0.495 | 0.455 | 0.686 | 0.160 | 0.294 | 0.488 | 0.493 | 0.580 |  | **0.057** | <0.01 |
|  | 9 | 0.516 | 0.514 | 0.538 | 0.493 | 0.668 | 0.185 | 0.303 | 0.466 | 0.475 | 0.557 | **0.008** |  | <0.01 |
|  | 10 | 0.669 | 0.649 | 0.674 | 0.578 | 0.456 | 0.427 | 0.251 | 0.303 | 0.319 | 0.452 | 0.393 | 0.386 |  |
|  |  |  |  |  |  |  |  |  |  |  |  |  |  |  |

| **Appendix Table 5** Results of one-tailed Wilcoxon tests conducted on microsatellite data of *Pyura doppelgangera* at 10 sites using the program BOTTLENECK. | | |
| --- | --- | --- |
| Site | *P* (H excess) | *P* (H deficiency) |
| 1 | 0.92 | 0.11 |
| 2 | 0.98 | 0.03 |
| 3 | 0.98 | 0.02 |
| 4 | 0.96 | 0.05 |
| 5 | 0.66 | 0.42 |
| 6 | 0.97 | 0.06 |
| 9 | 0.69 | 0.41 |
| 10 | 0.81 | 0.63 |

| **Appendix Table 6** *M*-ratios calculcated for *Pyura doppelgangera* data from eight sites using three different models. | | | | | | | |
| --- | --- | --- | --- | --- | --- | --- | --- |
| Site | Mean *M* | Model 1 | | Model 2 | | Model 3 | |
|  |  | *M*c | *M*c >  Mean *M* | *M*c | *M*c >  Mean *M* | *M*c | *M*c >  Mean *M* |
| 1 | 0.52 | 0.49 | No | 0.83 | Yes | 0.74 | Yes |
| 2 | 0.69 | 0.48 | No | 0.80 | Yes | 0.71 | Yes |
| 3 | 0.56 | 0.49 | No | 0.83 | Yes | 0.73 | Yes |
| 4 | 0.59 | 0.49 | No | 0.83 | Yes | 0.74 | Yes |
| 5 | 0.49 | 0.49 | No | 0.82 | Yes | 0.73 | Yes |
| 6 | 0.63 | 0.49 | No | 0.82 | Yes | 0.73 | Yes |
| 8 | 0.77 | 0.49 | No | 0.83 | Yes | 0.75 | No |
| 10 | 0.72 | 0.50 | No | 0.83 | Yes | 0.75 | Yes |
| Model 1 (most conservative, high occurrence of type II errors): *p*_g_ (proportion of multistep alleles) = 0.88, δ_g_ (mean size of multi-step mutations) = 2.8; Model 2 (least conservative, high occurrence of type I errors): *p*_g_ = 0.1, δ_g_ = 3.1; Model 3 (intermediate, recommended by Peery et al. 2012): *p*_g_ = 0.22, δ_g_ = 3.1. | | | | | | | |

| **Appendix Table 7** Mean estimates of four demographic parameters estimated in MSVAR. Values in brackets are 95% highest posterior density intervals. | | | | |
| --- | --- | --- | --- | --- |
| Population | *N*_0_ | *N*_A_ |  | *T* |
| 1 | 79  (11 – 535) | 65 163  (6 166 – 645 654) | 4.0 × 10^-4^  (1.4 × 10^-4^ – 1.3 × 10^-3^) | 1 340  (173 – 10 617) |
| 8+9 | 240  (30 – 1 862) | 20 417  (955 – 354 813) | 4.1 × 10^-4^  (1.4 × 10^-4^ – 1.2 × 10^-3^) | 2 649  (132 – 57 544) |
| 10 | 132  (7 – 1 871) | 57 280  (3 548 – 954 993) | 4.0 × 10^-4^  (1.3 × 10^-4^ – 1.2 × 10^-3^) | 1 259  (58 – 24 547) |
| 2 – 7 | 509  (88 – 2 877) | 36 308  (6 026 – 223 872) | 4.1 × 10^-4^  (1.3 × 10^-4^ – 1.2 × 10^-3^) | 1 072  (141 – 7 727) |
| *N*_0_ = present effective populations size, *N*_A_ = ancestral effective population size prior,  = mutation rate, *T* = time since population size change. Population numbers are the same as those used in Table 1 and Fig. 1. Parameters in the fourth row were estimated for a data set that included all Tasmanian sampling locations. | | | | |

| **Appendix Table 8** Pairwise estimates of divergence time (T) among Tasmanian populations of *Pyura doppelgangera*. For each pair of sites, three estimates of T (1, 2 and 3) are shown. HPD95Lo and HPD95Lo are lower and upper 95% highest posterior density intervals, respectively. Means (SD) of these estimates of T and their 95% highest posterior density intervals are shown in Table 6. | | | | | | | | | | |
| --- | --- | --- | --- | --- | --- | --- | --- | --- | --- | --- |
| Site 0 | Site 1 | T (1) | HPD95Lo | HPD95Hi | T (2) | HPD95Lo | HPD95Hi | T (3) | HPD95Lo | HPD95Hi |
| 1 | 3+4 | 5 | 0 | 72 | 3 | 0 | 42 | 4 | 0 | 57 |
| 8+9 | 3+4 | 2 | 0 | 39 | 2 | 0 | 35 | 2 | 0 | 27 |
| 10 | 3+4 | 3 | 0 | 29 | 1 | 0 | 25 | 3 | 0 | 25 |
|  |  |  |  |  |  |  |  |  |  |  |
| 2 | 3 | 12 | 2 | 428 | 19 | 2 | 427 | 11 | 2 | 423 |
|  | 4 | 15 | 2 | 453 | 16 | 0 | 502 | 24 | 0 | 367 |
|  | 5 | 111 | 2 | 890 | 105 | 7 | 853 | 91 | 2 | 823 |
|  | 6 | 270 | 58 | 1165 | 330 | 0 | 1128 | 328 | 44 | 1154 |
|  | 7 | 213 | 58 | 965 | 208 | 43 | 938 | 216 | 37 | 942 |
| 3 | 4 | 23 | 3 | 179 | 25 | 2 | 172 | 8 | 1 | 138 |
|  | 5 | 50 | 4 | 350 | 39 | 5 | 296 | 23 | 1 | 299 |
|  | 6 | 63 | 8 | 351 | 62 | 5 | 396 | 63 | 5 | 344 |
|  | 7 | 131 | 28 | 651 | 155 | 19 | 664 | 127 | 16 | 585 |
| 4 | 5 | 69 | 8 | 413 | 65 | 0 | 392 | 61 | 5 | 392 |
|  | 6 | 19 | 0 | 541 | 11 | 0 | 481 | 13 | 0 | 470 |
|  | 7 | 163 | 32 | 756 | 158 | 21 | 726 | 156 | 29 | 741 |
| 5 | 6 | 23 | 0 | 257 | 25 | 0 | 300 | 17 | 0 | 244 |
|  | 7 | 224 | 13 | 1129 | 195 | 3 | 1094 | 156 | 4 | 860 |
| 6 | 7 | 398 | 11 | 1453 | 502 | 0 | 1456 | 421 | 23 | 1538 |
|  |  |  |  |  |  |  |  |  |  |  |

**Appendix Figure legends**

**Appendix Figure 1** Maximum-likelihood bootstrap trees of a) COI sequences and b) ANT intron sequences. Sites 1-10 are listed in Table 2. Samples from several additional sites are included for which no microsatellite data were generated due to low sample sizes or low sample quality: Beauty Point (41°09'S 146°49'E), Coles Bay (42°07'S 148°17'E), Taroona (42°57'S 147°21'E), Two Tree Point (43°20'S 147°19'E) and Cowes (38°26'S 145°14'E). Nucleotide evolution models (the Tamura-Nei model for COI, Tamura and Nei 1993, and the Jukes-Cantor model, Jukes and Cantor 1969, for ANT) were estimated using the Bayesian Information Criterion in MEGA v.5 (Tamura et al. 2011).

**Appendix Figure 2** Factorial correspondence analysis (FCA) plots using microsatellite data from 10 populations of *Pyura doppelgangera* from Tasmania, South Australia, Victoria and New Zealand. Colours represent to the following populations: 1. South Australia = Yellow, 2. Trial Harbour = Blue, 3. Couta Rocks = White, 4. Bridport = Grey, 5. The Gardens = Pink, 6. Bicheno = Turquoise, 7. Pirates Bay = Dark blue, 8. Port Welshpool = Marroon, 9. Port Albert = Green, 10. New Zealand = Brown. Western Tasmania includes sites 2 and 3, Northern Tasmania comprises site 4, Eastern Tasmania consists of sites 5, 6 and 7, and Victoria includes sites 8 and 9 (Table 2).

**Appendix Figure 3** Estimation of the number of clusters (*K*) in the reduced data set of *Pyura doppelgangera* microsatellites; a) Mean likelihood of each value of *K* based on three replications; b) Δ*K* (rate of change in likelihoods between successive values of *K*).
