# Supplementary material for: Can novel genetic analyses help to identify low-dispersal marine invasive species?
Source: Ecol Evol. 2014 Jun 24;4(14):2848–66. doi: 10.1002/ece3.1129 (PMC4130444; doi:10.1002/ece3.1129)
Supplement: Supplementary file 2 — Figure S1. Maximum-likelihood bootstrap trees of (a) COI sequences and (b) ANT intron sequences. [file ece30004-2848-SD2.pdf]

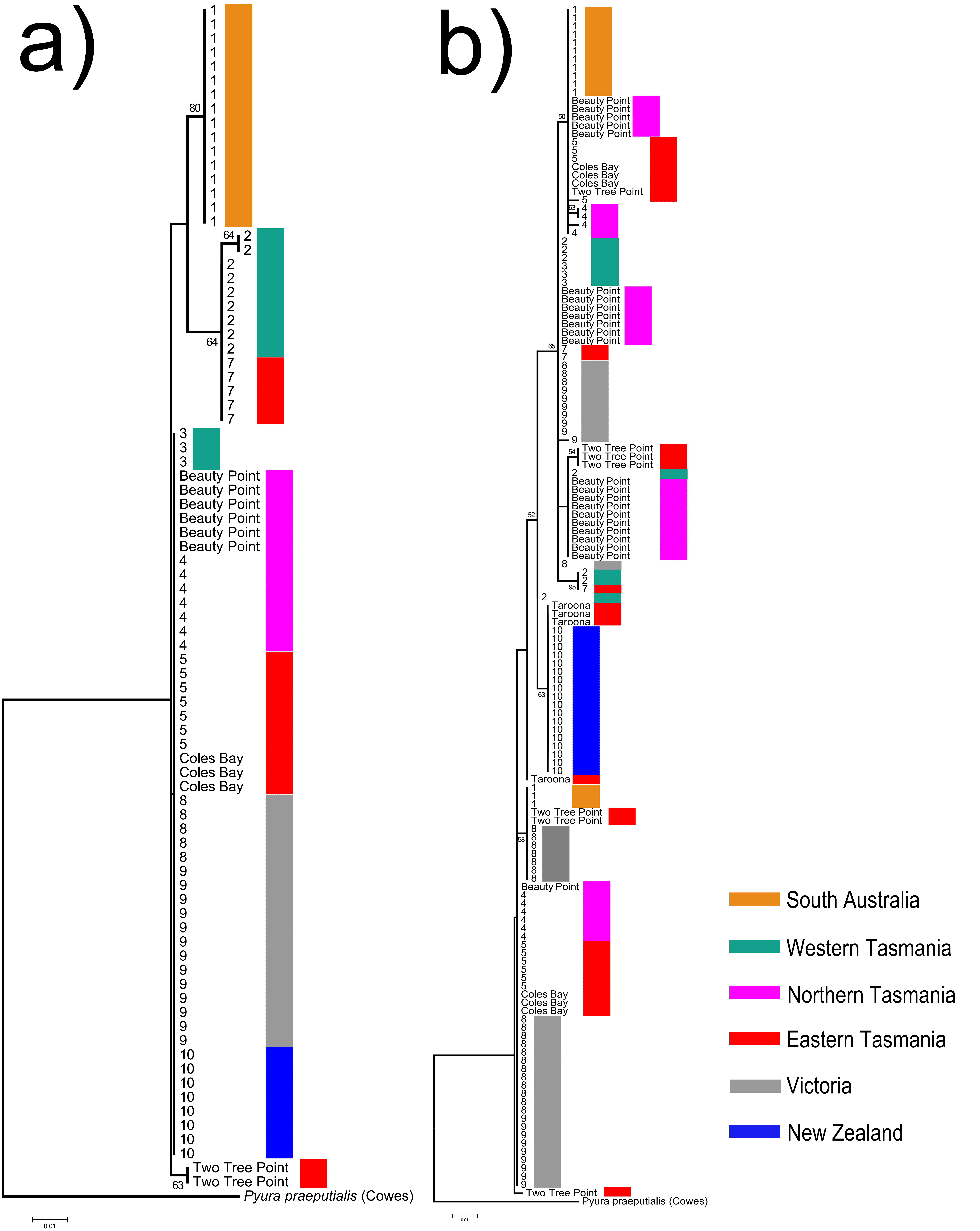
a)
b)
80
64
64
63
50
63
65
54
52
95
63
58
Beauty Point
Coles Bay
Two Tree Point
Taroona
Pyura praeputialis (Cowes)
0.01
South Australia
Western Tasmania
Northern Tasmania
Eastern Tasmania
Victoria
New Zealand
