# Supplementary figures and images for: Can novel genetic analyses help to identify low-dispersal marine invasive species?
Source: Ecol Evol. 2014 Jun 24;4(14):2848–66. doi: 10.1002/ece3.1129 (PMC4130444; doi:10.1002/ece3.1129)

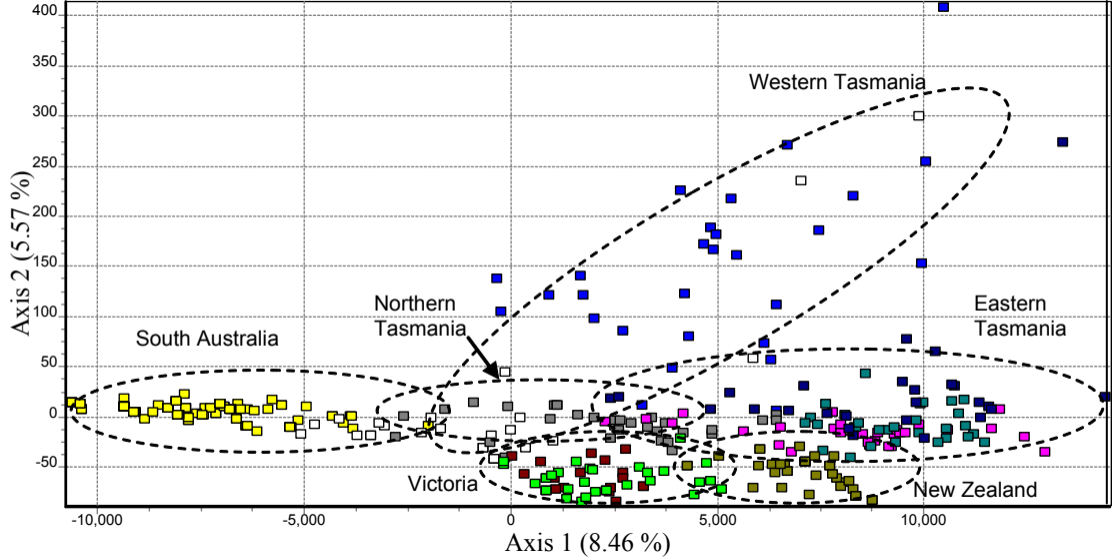

Supplement: Supplementary file 3 — Figure S2. Factorial correspondence analysis (FCA) plots using microsatellite data from 10 populations of Pyura doppelgangera from Tasmania, South Australia, Victoria, and New Zealand. [file ece30004-2848-SD3.pdf]

a)

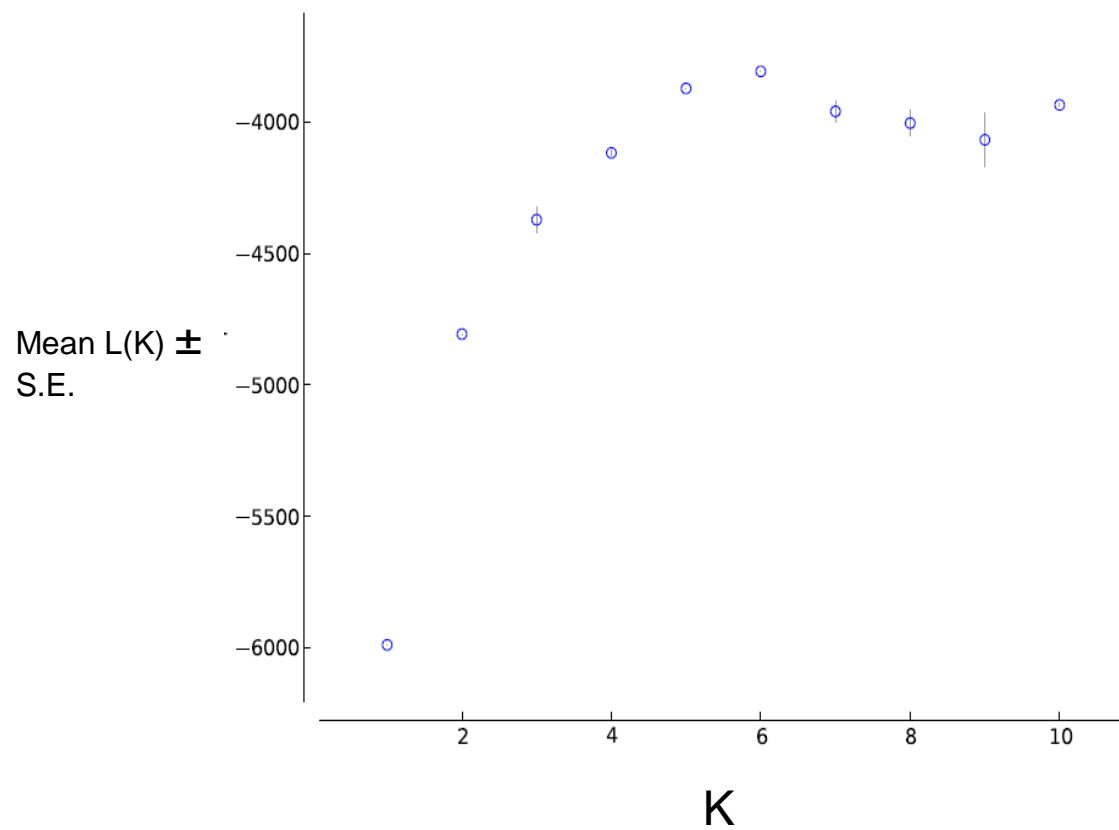

b)

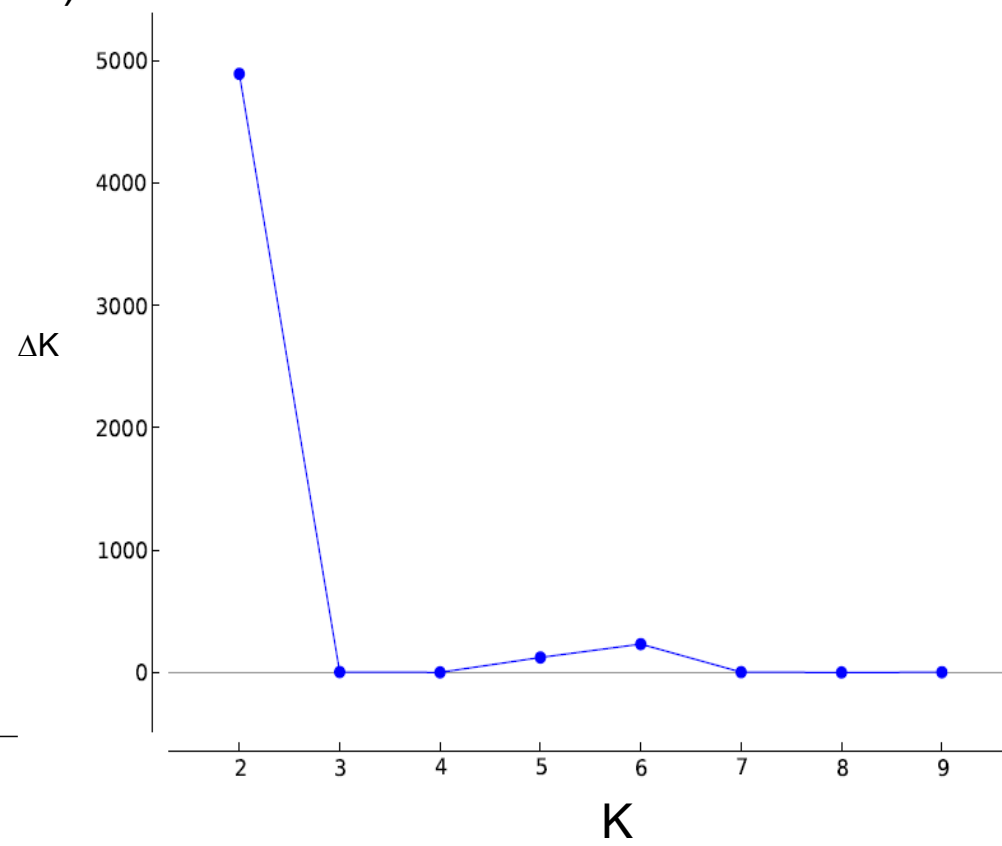

Supplement: Supplementary file 4 — Figure S3. Estimation of the number of clusters (K) in the reduced data set of Pyura doppelgangera microsatellites. [file ece30004-2848-SD4.pdf]
